# Sugar-Sweetened Beverage Intake and Cancer Recurrence and Survival in CALGB 89803 (Alliance)

**DOI:** 10.1371/journal.pone.0099816

**Published:** 2014-06-17

**Authors:** Michael A. Fuchs, Kaori Sato, Donna Niedzwiecki, Xing Ye, Leonard B. Saltz, Robert J. Mayer, Rex B. Mowat, Renaud Whittom, Alexander Hantel, Al Benson, Daniel Atienza, Michael Messino, Hedy Kindler, Alan Venook, Shuji Ogino, Kana Wu, Walter C. Willett, Edward L. Giovannucci, Jeffrey A. Meyerhardt

**Affiliations:** 1 Brown University, Providence, Rhode Island, United States of America; 2 Dana-Farber Cancer Institute, Boston, Massachusetts, United States of America; 3 Alliance Statistics and Data Center, Duke University Medical Center, Durham, North Carolina, United States of America; 4 Memorial Sloan-Kettering Cancer Center, New York, New York, United States of America; 5 Toledo Community Hospital Oncology Program, Toledo, Ohio, United States of America; 6 Hôpital du Sacré-Coeur de Montréal, Montreal, Quebec, Canada; 7 Loyola University Stritch School of Medicine, Naperville, Illinois, United States of America; 8 Robert H. Lurie Comprehensive Cancer Center, Northwestern University, Chicago, Illinois, United States of America; 9 Virginia Oncology Associates, Norfolk, Virginia, United States of America; 10 Southeast Cancer Control Consortium, Mission Hospitals, Inc., Asheville, North Carolina, United States of America; 11 University of Chicago, Chicago, Illinois, United States of America; 12 University of California at San Francisco Comprehensive Cancer Center, San Francisco, California, United States of America; 13 Department of Pathology, Brigham and Women’s Hospital, Boston, Massachusetts, United States of America; 14 Department of Nutrition, Harvard School of Public Health, Boston, Massachusetts, United States of America; 15 Department of Epidemiology, Harvard School of Public Health, Boston, Massachusetts, United States of America; 16 Channing Laboratory, Department of Medicine, Brigham and Women’s Hospital, and Harvard Medical School, Boston, Massachusetts, United States of America; University of Bari & Consorzio Mario Negri Sud, Italy

## Abstract

**Background:**

In colon cancer patients, obesity, sedentary lifestyle, and high dietary glycemic load have been associated with increased risk of cancer recurrence. High sugar-sweetened beverage intake has been associated with obesity, diabetes, and cardio-metabolic diseases, but the influence on colon cancer survival is unknown.

**Methods:**

We assessed the association between sugar-sweetened beverage consumption on cancer recurrence and mortality in 1,011 stage III colon cancer patients who completed food frequency questionnaires as part of a U.S. National Cancer Institute-sponsored adjuvant chemotherapy trial. Hazard ratios (HRs) and 95% confidence intervals (CIs) were calculated with Cox proportional hazard models.

**Results:**

Patients consuming ≥2 servings of sugar-sweetened beverages per day experienced an adjusted HR for disease recurrence or mortality of 1.67 (95% CI, 1.04–2.68), compared with those consuming <2 servings per month (*P*_trend_ = 0.02). The association of sugar-sweetened beverages on cancer recurrence or mortality appeared greater among patients who were both overweight (body mass index ≥25 kg/m^2^) and less physically active (metabolic equivalent task-hours per week <18) (HR = 2.22; 95% CI, 1.29–3.81, *P*_trend_ = 0.0025).

**Conclusion:**

Higher sugar-sweetened beverage intake was associated with a significantly increased risk of cancer recurrence and mortality in stage III colon cancer patients.

## Introduction

Increasing evidence indicates that excess energy balance, including obesity, sedentary lifestyle, and high intake of a Western pattern diet, is associated with an increased risk of developing colon cancer. [1]–[4] One hypothesis explaining these observations has been the role of hyperinsulinemia in promoting cancer development and progression. [5], [6] Prospective studies demonstrate associations between colon cancer risk among individuals with diabetes or elevated circulating levels of insulin or C-peptide. [6]–[8] Beyond cancer risk, observational studies of colon cancer patients suggest that these same host factors, including obesity, sedentary lifestyle, high intake of a Western pattern diet, and high dietary glycemic load confer an increased risk of colon cancer recurrence [9]–[11].

Across numerous studies, higher intake of sugar-sweetened beverages has been associated with an increased risk of obesity, type 2 diabetes, and related cardio-metabolic diseases. [12]–[17] Consumption of sugar-sweetened beverages has increased in the U.S. over the past 3 decades; per capita consumption in 2009 was estimated at 45 gallons per year, or nearly half of total beverage intake. [18] Worldwide, sugar-sweetened beverages have been associated with 180,000 deaths annually. [19] Nonetheless, the influence of sugar-sweetened beverage intake on colon cancer risk or colon cancer patient outcomes remains largely unknown.

In light of the emerging evidence supporting a link between excess energy balance, hyperinsulinemia and inferior survival in colon cancer patients, we prospectively examined the impact of sugar-sweetened beverage intake on cancer recurrence and mortality in a cohort of stage III colon cancer patients enrolled in a National Cancer Institute-sponsored adjuvant chemotherapy clinical trial. We prospectively collected extensive data on diet and lifestyle prior to any documentation of cancer recurrence.

## Materials and Methods

### Study Population

Patients in this prospective cohort participated in the National Cancer Institute–sponsored Cancer and Leukemia Group B (CALGB, now Alliance for Clinical Trials in Oncology) 89803 adjuvant therapy trial for stage III colon cancer, comparing therapy with weekly 5-fluorouracil and leucovorin to weekly irinotecan, 5-fluorouracil, and leucovorin (ClinicalTrials.gov NCT000038350). [20] Between May 1999 and May 2001, 1,264 patients were enrolled in the treatment trial. After 87 patients were enrolled, an amendment required patients to complete a self-administered questionnaire that captured diet and lifestyle habits midway through their therapy (4 months after surgery; questionnaire 1 [Q1]) and again 6 months after completion of treatment (14 months after surgery; Q2).

**Figure 1:**
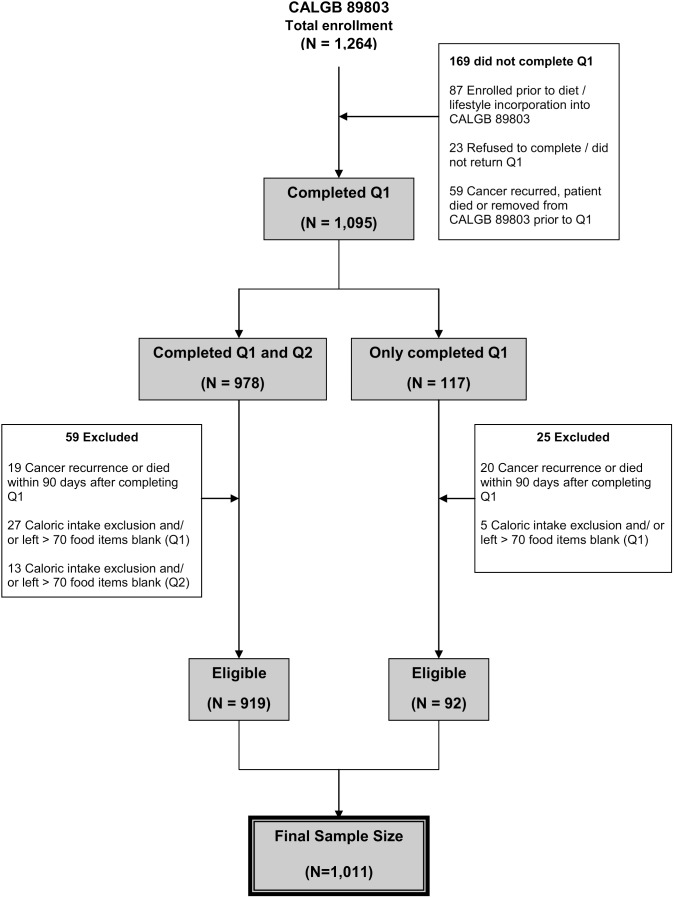
Derivation of the study cohort. Q1 = question 1 (midway through adjuvant therapy); Q2 = questionnaire 2 (6 months after completion of adjuvant therapy). Caloric intake exclusion = Less than 600 calories or greater than 4,200 calories per day for men and less than 500 calories or greater than 3,500 calories per day for women.

Patients were eligible if they underwent a complete surgical resection of the primary tumor within 56 days of trial entry, had regional lymph node metastases but no evidence of distant metastases, had a baseline Eastern Cooperative Oncology Group performance status of 0 to 2, [21] and had adequate bone marrow, renal, and hepatic function. **Figure 1** illustrates the derivation of the final sample size of 1,011 patients for this study. We previously demonstrated that there were no appreciable differences in baseline characteristics between the 1,011 patients who were eligible for this analysis and the remaining 253 patients enrolled in CALGB 89803 not included in the dietary analysis. [22] All patients signed informed consent, which was approved by the National Cancer Institute’s Cancer Treatment Evaluation Program (CTEP) as well as each participating site’s institutional review board (IRB), including the Dana-Farber/Harvard Cancer Center IRB.

### Dietary Assessment

Patients in this analysis completed semi-quantitative food frequency questionnaires (FFQs) that included 131 food items, vitamin and mineral supplements, and open-ended sections for other supplements and foods not specifically listed. [23], [24] Participants were asked how often, on average over the previous 3 months, they consumed a specific food portion size, with up to nine possible responses, which ranged from never to six or more times per day. We computed nutrient intakes by multiplying the frequency of consumption of each food by the nutrient content of the specified portions using composition values from Department of Agriculture sources supplemented with other data [25] All nutrient values were energy-adjusted using the residuals methods [26].

On each FFQ, patients reported on their usual daily or weekly intake of a standard 355-mL (12-ounce) serving (1 glass, can, or bottle) of sugar-sweetened caffeinated colas, caffeine-free colas, other carbonated sugar-sweetened beverages, and noncarbonated sugar-sweetened beverages (fruit punches, lemonades, or other fruit drinks). We summed the consumption of these beverages as total sugar-sweetened beverages. In other cohort studies, the reproducibility and validity of our FFQ in measuring sugar-sweetened beverages was previously described: the Pearson correlation coefficient, corrected for within-person variation, between the FFQ and separate dietary records was 0.84 for cola and 0.36–0.40 for non-cola soda [27] In addition, we separately validated this FFQ among cancer patients receiving chemotherapy [28].

Patients who completed Q1 and whose cancer had not recurred prior to Q1 completion were included in these analyses. The median time from study entry to Q1 was 3.5 months (95% range**,** 2.5–5.0 months; full range**,** 0.2–9.9 months). To avoid potential biases due to declining health immediately before cancer recurrence or death, we excluded patients who experienced cancer recurrence or death within 90 days following completion of Q1 (Figure 1). We calculated dietary exposures based on the results of the Q2 using cumulative averaging as previously described,[29] but weighted proportional to times between Q1 and Q2 and then between Q2 and disease-free survival time. For example, if a patient completed Q1 at 4 months, completed Q2 at 14 months, and had a cancer recurrence at 30 months, the total time between Q1 and cancer recurrence was 26 months and 38% of that time was between Q1 and Q2 and 62% of that time was between Q2 and the recurrence.

### Study Endpoints

The primary end point of our study was disease-free survival, defined as time from the completion of the Q1 to tumor recurrence, occurrence of a new primary colon tumor, or death from any cause. We also assessed recurrence-free survival, defined as the time from the completion of Q1 to tumor recurrence or occurrence of a new primary colon tumor. For recurrence-free survival, patients who died without known tumor recurrence were censored at last documented evaluation by treating physician. Finally, overall survival was defined as the time from the completion of Q1 to death from any cause.

### Statistical Analysis

Sugar-sweetened beverage intake was classified by frequency of 12-ounce servings: less than 2 per month (reference), 2 per month to 2 per week, 3 to 6 per week, 1 per day to less than 2 per day, and 2 or more per day. Cox proportional hazards regression was used to determine the simultaneous impact of other variables potentially associated with each outcome. [30] As previously described, [26] all analyses of sugar-sweetened beverage intake were adjusted for total caloric intake to assess the effect of sugar-sweetened beverages independent of total energy intake. We further used time-varying covariates to adjust for total calories, physical activity (measured in metabolic equivalent tasks [MET]-hours per week), [31] body mass index (BMI, calculated as weight in kilograms divided by height in meters squared), and consumption of Western and prudent pattern diets [10] with updating from the second questionnaire. Other covariates (including age at study entry, gender, number of positive lymph nodes, depth of invasion through bowel wall, baseline performance status, and treatment group) were also entered into the model as fixed covariates. In secondary multivariate analyses, we further adjusted for dietary glycemic load. [22] Covariates with missing variables were coded with indicator variables in adjusted models. We tested for linear trends across categories of sugar-sweetened beverages by assigning each participant the median value for each category and modeling this value as a continuous variable, consistent with prior studies. [10], [22] In subgroup exploratory analyses, the five intake categories were collapsed (1 and 2; 3 and 4; 5) to conserve power to create 3 groupings with practical meaning – less than twice a week, several times per week to daily and more than 2 per day. The Cox regression models met the assumption of proportionality. A level of significance <0.05 was considered statistically significant. All *P* values are 2-sided and were not adjusted for multiple comparisons.

Patient registration and clinical data collection were managed and analyses were conducted by the Alliance Statistics and Data Center. All analyses were based on the study database frozen on November 9, 2009.

## Results

Study participants were drawn from a multicenter study of adjuvant chemotherapy after surgery in patients with stage III colon cancer. Baseline characteristics by level of sugar-sweetened beverage consumption are shown in **Table 1**. Patients consuming greater quantities of sugar-sweetened beverages were younger and more likely to be male and had a higher intake of a Western pattern diet and dietary glycemic load.

**Table 1:** Baseline characteristics of 1,011 patients by consumption level of sugar-sweetened beverage consumption*.

	Consumption level				
	<2/mo	2/mo to 2/wk	3 to 6/wk	1 to <2/d	≥2/d
	(n = 212)	(n = 355)	(n = 258)	(n = 115)	(n = 71)
Median	0.5/mo	5.5/mo	4.6/wk	1.3/d	2.6/d
Age, median (range), years	63.0 (32–85)	63.0 (28–83)	58.0 (24–81)	57.0 (21–80)	51.0 (27–74)
Male, No (%)	93 (43.9)	187 (52.7)	162 (62.8)	72 (62.6)	55 (77.5)
Race, No (%)					
White	195 (92.0)	323 (91.0)	226 (87.6)	97 (84.3)	58 (81.7)
Black	5 (2.3)	16 (4.5)	19 (7.4)	14 (12.2)	11 (15.5)
Other	12 (5.7)	16 (4.5)	13 (5.0)	4 (3.5)	2 (2.8)
Baseline performance status, No. (%)‡					
0	157 (74.1)	263 (74.1)	194 (75.2)	79 (68.7)	49 (69.0)
1–2	49 (23.1)	86 (24.2)	61 (23.6)	31 (27.0)	21 (29.6)
Status unknown	6 (2.8)	6 (1.7)	3 (1.2)	5 (4.3)	1 (1.4)
Invasion through bowel wall by T stage, No. (%)¥					
T1–2	29 (13.7)	54 (15.2)	29 (11.2)	13 (11.3)	11 (15.5)
T3–4	169 (79.7)	283 (79.7)	210 (81.4)	89 (77.4)	56 (78.9)
T stage unknown	14 (6.6)	18 (5.1)	19 (7.4)	13 (11.3)	4 (5.6)
Positive lymph nodes, No. (%)					
1–3	141 (66.5)	229 (64.5)	166 (64.3)	58 (50.4)	41 (57.8)
≥4	65 (30.7)	121 (34.1)	90 (34.9)	52 (45.2)	28 (39.4)
Nodes unknown	6 (2.8)	5 (1.4)	2 (0.8)	5 (4.4)	2 (2.8)
Grade of differentiation, No. (%)					
Well	18 (8.5)	13 (3.6)	17 (6.6)	5 (4.3)	4 (5.7)
Moderate	140 (66.0)	259 (73.0)	178 (69.0)	75 (65.3)	49 (69.0)
Poor/Undifferentiated	48 (22.7)	76 (21.4)	61 (23.6)	30 (26.1)	17 (23.9)
Grade unknown	6 (2.8)	7 (2.0)	2 (0.8)	5 (4.3)	1 (1.4)
Clinical bowel obstruction at presentation, No. (%)	43 (20.3)	77 (21.7)	56 (21.7)	29 (25.2)	17 (23.9)
Bowel perforation at presentation, No. (%)	9 (4.2)	12 (3.4)	17 (6.6)	2 (1.7)	3 (4.2)
Treatment arm, No. (%)					
5-FU/LV	104 (49.1)	180 (50.7)	136 (52.7)	57 (49.6)	36 (50.7)
IFL	108 (50.9)	175 (49.3)	122 (47.3)	58 (50.4)	35 (49.3)
Body mass index, median (range), kg/m^2^ § †	27.0 (17–50)	27.2 (18–52)	27.4 (16–49)	27.3 (16–46)	28.0 (17–50)
Physical activity, median (range) MET h/wk§ ^□^	4.6 (0–125)	4.1 (0–90)	6.0 (0–125)	6.3 (0–147)	4.2 (0–113)
Western dietary pattern, No. <median (%)§	81 (38.2)	170 (47.9)	147 (57.0)	80 (69.6)	57 (80.3)
Prudent pattern diet, No. <median, (%)§	134 (63.2)	191 (53.8)	113 (43.8)	44 (38.3)	30 (42.3)
Dietary glycemic load §	138.2 (58–221)	143.0 (75–232)	147.2 (89–211)	163.4 (94–247)	168.3 (123–231)

Mo = month; No. = number; 5-FU = 5-fluorouracil; IFL = irinotecan, 5-fluorouracil, leucovorin; LV = leucovorin; MET = metabolic equivalent tasks.

‡ Baseline performance status: PS 0 = fully active; PS 1 = restricted in physically strenuous activity but ambulatory and able to carry out light work; PS 2 = ambulatory and capable of all self-care but unable to carry out any work activities, up and about more than 50% of waking hours.

¥ T1–2 = level of invasion through the bowel wall not beyond the muscle layer; T3–4 = level of invasion through the bowel wall beyond the muscle layer.

§ Based on questionnaire 1.

† 1 subject is missing body mass index in questionnaire 1.

□ 6 subjects are missing physical activity in quesitonnaire 1.

The median follow-up from the time of completion of Q1 was 7.3 years. In total, 343 of the 1,011 patients included in this analysis had cancer recurrence; 262 of these 343 patients died. An additional 43 patients died without documented cancer recurrence.

The pre-defined primary endpoint of our analysis was disease-free survival. Increasing consumption of sugar-sweetened beverages was associated with a greater risk of cancer recurrence or mortality after adjusting for other predictors of cancer recurrence (**Table 2**). Compared to patients who drank less than two sugar-sweetened beverage per month, consumers of 2 or more servings per day experienced an adjusted hazard ratio (HR) for disease recurrence or mortality of 1.67 (95% confidence interval [CI] = 1.04–2.68; *P*_trend_ = 0.02). These results were largely unchanged when we further adjusted for dietary glycemic load in the multivariate model (*P*_trend_ = 0.03).

**Table 2:** Associations between colon cancer recurrence and mortality and consumption level of sugar-sweetened beverages.

Outcome		Consumption level					
		<2/mo	2/mo to 2/wk	3 to 6/wk	1 to <2/d	≥2/d	*P*_trend_ *
		(n = 212)	(n = 355)	(n = 258)	(n = 115)	(n = 71)	HR (95% CI)
Disease-free survival							
No. of events for energy-adjusted model		82	121	108	43	32	
Energy adjusted only, HR (95% CI)		1.0	0.87	1.17	1.02	1.46	0.03
			(0.66–1.16)	(0.88–1.57)	(0.70–1.48)	(0.95–2.25)	1.18 (1.02–1.36)
Multivariable adjusted, HR (95% CI)‡		1.0	0.88	1.23	1.03	1.67	0.02
			(0.66–1.17)	(0.90–1.66)	(0.69–1.51)	(1.04–2.68)	1.22 (1.04–1.44)
Multivariable adjusted, HR (95% CI)§		1.0	0.87	1.21	1.01	1.62	0.03
			(0.66–1.16)	(0.89–1.65)	(0.67–1.50)	(0.99–2.65)	1.21 (1.02–1.44)
Recurrence-free survival							
No. of events for energy-adjusted model		66	110	99	38	30	
Energy adjusted only, HR (95% CI)		1.0	0.99	1.33	1.12	1.70	0.01
			(0.73–1.34)	(0.97–1.83)	(0.75–1.68)	(1.08–2.67)	1.21 (1.04–1.41)
Multivariable adjusted, HR (95% CI)‡		1.0	0.99	1.37	1.11	1.84	0.02
			(0.73–1.35)	(0.98–1.89)	(0.73–1.69)	(1.12–3.04)	1.24 (1.04–1.47)
Multivariable adjusted, HR (95% CI)§		1.0	0.98	1.34	1.07	1.75	0.04
			(0.72–1.34)	(0.97–1.87)	(0.70–1.65)	(1.04–2.94)	1.22 (1.01–1.46)
Overall survival							
No. of events for energy-adjusted model		69	95	84	30	27	
Energy adjusted only, HR (95% CI)		1.0	0.78	1.07	0.76	1.26	0.29
			(0.56–1.09)	(0.76–1.51)	(0.48–1.22)	(0.76–2.10)	1.10 (0.92–1.32)
Multivariable adjusted, HR (95% CI)‡		1.0	0.75	1.09	0.74	1.51	0.12
			(0.54–1.05)	(0.77–1.55)	(0.45–1.19)	(0.87–2.63)	1.17 (0.96–1.43)
Multivariable adjusted, HR (95% CI)§		1.0	0.74	1.07	0.70	1.41	0.21
			(0.53–1.04)	(0.75–1.53)	(0.43–1.15)	(0.79–2.50)	1.14 (0.93–1.41)

HR = hazard ratio; CI = confidence interval; mo = month; wk = week; d = day.

*Two-sided P value. Trend across consumption levels.

‡ Adjusting with Cox proportional hazards regression for age, sex, depth of invasion through bowel wall, number of positive lymph nodes, baseline performance status, treatment group, and the following time-varying covariates: total energy intake, body mass index, physical activity level, Western dietary pattern, and prudent dietary pattern.

§ Adjusting for all above and time-varying dietary glycemic load.

To isolate the influence of sugar-sweetened beverage intake on cancer recurrence, we assessed the endpoint of recurrence-free survival and confirmed that higher intake of sugar-sweetened beverage conferred a significantly increased risk in cancer recurrence (*P*_trend_ = 0.02; **Table 2**). Patients consuming 2 or more servings per day were 1.84 times more likely to recur than those who largely abstained. We observed a trend toward greater overall mortality with consumption of 2 or more sugar-sweetened beverages per day, although the results were not statistical significant (*P*_trend_ = 0.12).

Taking into account the possibility that dietary changes could reflect occult cancer or imminent death, we excluded patients who developed cancer recurrence or died within 90 days of completing Q1 in our initial analyses. To further explore the potential influence of occult cancer or imminent death, we excluded 44 patients who developed cancer or died within 180 days of completing Q1; our results remained largely unchanged. Increasing sugar-sweetened beverage intake was associated with an inferior disease-free survival (*P*_trend_ = 0.04) and an inferior recurrence-free survival (*P*_trend_ = 0.04).

We explored whether the association between sugar-sweetened beverage intake and patient outcome differed between individual sugar-sweetened beverages subtypes (colas; other carbonated beverages; and non-carbonated fruit drink and punches). The association between consumption of specific sugar-sweetened beverages and survival endpoints did not differ between beverage subtypes; tests for heterogeneity between subtypes was not statistically significant for disease-free survival (*P* = 0.65), recurrence-free survival (*P* = 0.59), or overall survival (*P* = 0.71), though power for these analyses is limited.

**Figure 2:**
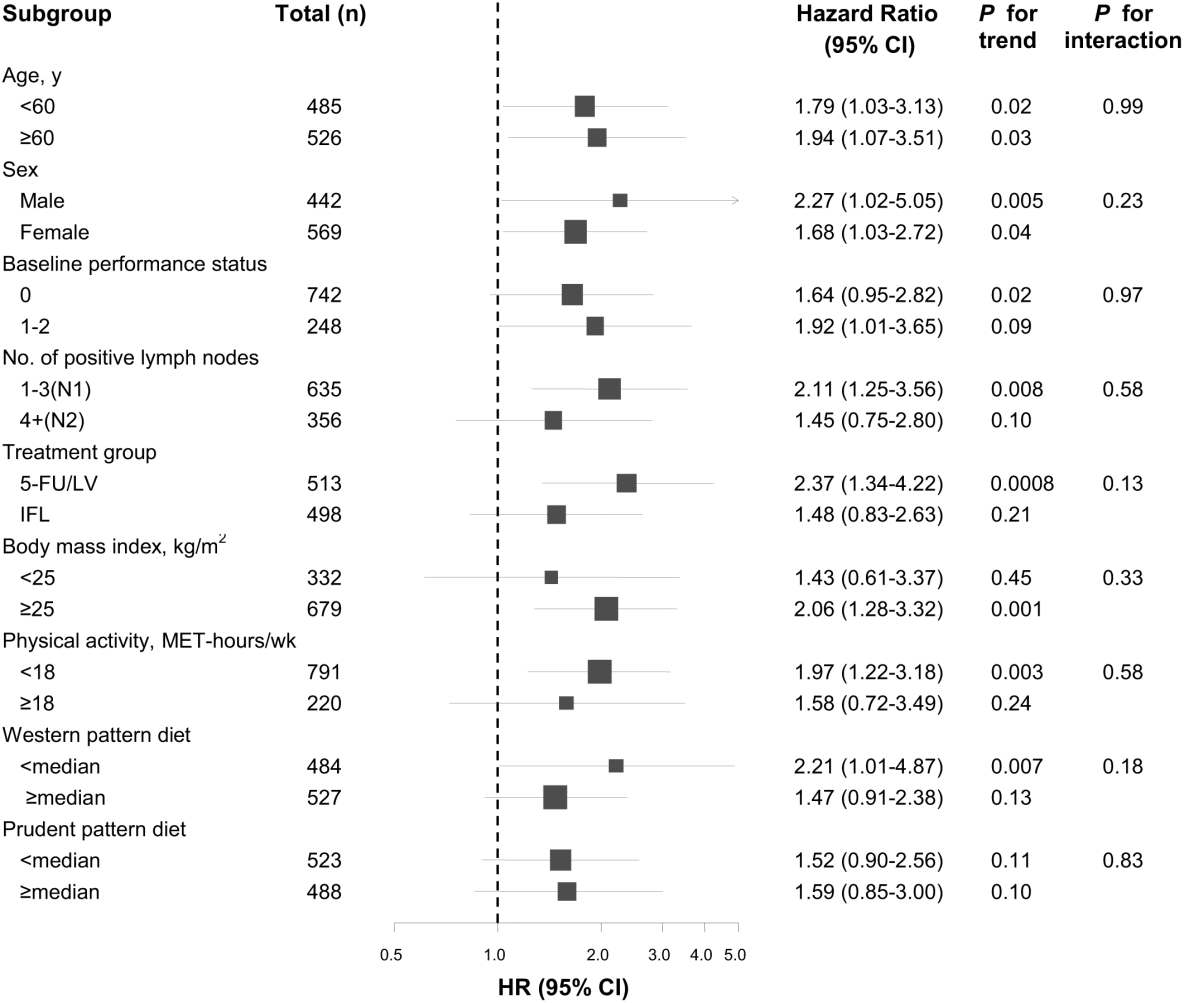
Multivariate hazard ratios and 95% CI for cancer recurrence and mortality across strata of various factors. Analyses utilized 3 categories (2 or less servings per week, between 3 servings per week and less than 2 servings per day and 2 or more serving per day. The forest plot represents the hazard ratios of the comparison of ≥2 servings per day of sugar-sweetened beverage intake to ≤2 serving per week. No. = Number; y = years; kg = kilograms; m = meters; wk = weeks; 5-FU/LV = 5-fluorouracil/leucovorin; IFL = bolus 5-fluorouracil, leucovorin, and irinotecan.

We also examined the influence of sugar-sweetened beverage intake on disease-free survival across strata of other potential predictors of patient outcome (**Figure 2**), using three categories of intake (2 or less beverages per week, between 3 beverages per week and less than 2 per day and 2 or more beverages per week). In the forest plot, we demonstrate the hazard ratio comparing the two extreme categories. The association of sugar-sweetened beverage intake was not significantly modified by any potential predictors of patient outcome.

**Figure 3:**
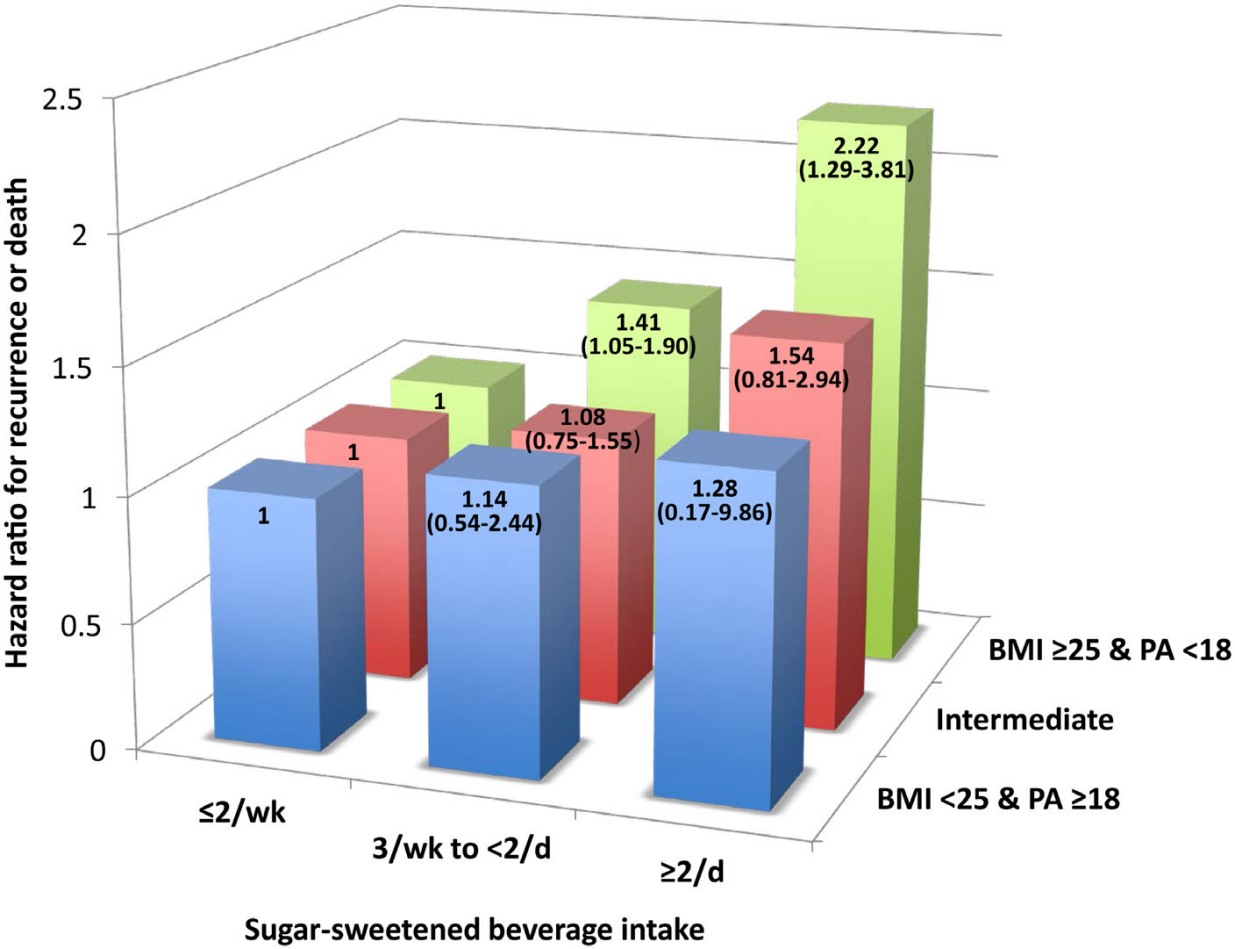
Multivariate hazard ratios for cancer recurrence or death according to combinations of body mass index, physical activity, and sugar-sweetened beverage intake (3 categories). BMI = body mass index in kg/m^2^; PA = physical activity in MET-hours per week; wk = week, d = day. Intermediate = BMI≥25 kg/m^2^ and PA≥18 MET-hours per week or BMI<25 kg/m^2^ and PA<18 MET-hours per week.

We further explored whether the association between sugar-sweetened beverage intake and the risk for cancer recurrence or mortality was more pronounced among patients who were both overweight (BMI≥25 kg/m^2^) and less physically active (<18 MET-hours/week) (**Figure 3**). Among patients who were both overweight and less active (n = 541), the adjusted HR for cancer recurrence or mortality was 2.22 (95% CI, 1.29–3.81) when comparing the highest and the lowest categories of sugar-sweetened beverage intake (*P*_trend_ = 0.0025). In contrast, among the 84 patients who were both normal weight (<25 kg/m^2^) and physically active (MET-hours per week ≥18), sugar-sweetened beverage intake did not significantly influence the risk of cancer recurrence or mortality (*P*_trend_ = 0.67). A test for statistical interaction between sugar-sweetened beverage intake and the combined assessment of BMI and physical activity was, however, not significant (*P*_interaction_ = 0.40).

## Discussion

In this cohort of stage III colon cancer patients enrolled in a clinical trial of postoperative adjuvant chemotherapy, increased intake of sugar-sweetened beverages was associated with a significantly increased risk of cancer recurrence or mortality, and the deleterious association of sugar-sweetened beverages was largely confined to patients who consumed 2 or more servings per day. The association between sugar-sweetened beverage consumption and patient outcome was independent of other measures of energy balance, including body-mass index, Western pattern diet, and dietary glycemic load.

To our knowledge, no previous study has examined the association of sugar-sweetened beverage intake on clinical outcome in patients with established colon cancer. Two studies have examined the relation between sugar-sweetened beverage intake and the risk of developing colon cancer, and the results have been inconsistent. [32], [33] In otherwise healthy cohorts, sugar-sweetened beverages have been linked with weight gain, diabetes, hypertension, hyperlipidemia, gout, and coronary artery disease. [13]–[17], [34], [35] In randomized clinical trials, replacement of sugar-containing beverage with a sugar-free beverage significantly reduced weight gain and fat accumulation in children and adolescents. [36], [37] In a recent observational study of healthy adults, the association between sugar-sweetened beverage and obesity was significantly greater in subjects with a genetic predisposition to obesity. [38] Interestingly, sugar-sweetened beverage intake was associated with significant increase in cancer recurrence and mortality among patients who were both overweight and less physically active, whereas there did not appear to be an association among patients who were normal weight and physically active.

Previous observational studies of colon cancer patients have observed increased risks of cancer recurrence or mortality in association with a history of type 2 diabetes, obesity, sedentary lifestyle, high Western pattern diet, and greater dietary glycemic load. [9]–[11] While the mechanisms accounting for these observations remain uncertain, chronic hyperinsulinemia, promoting increased cancer cell proliferation and reduced apoptosis, has been proposed as one putative explanation. [39]–[42] In support of the hypothesis, higher circulating C-peptide was associated with increased cancer mortality in a population of stage I–III colorectal cancer patients [11].

Sugar-sweetened beverages provide a high caloric content with low satiety and incomplete compensation for these liquid calories, resulting in an increased total energy intake. [43], [44] In addition, because of the large amounts of rapidly absorbable carbohydrates in sugar-sweetened beverages, greater consumption may increase the risks of insulin resistance, beta-cell dysfunction, inflammation, visceral adiposity, and other metabolic disorders. [13], [45] In a prior analysis of our colon cancer patient cohort, neither total sucrose nor fructose intake was significantly associated with patient outcome. [22] Nonetheless, sucrose or fructose from beverages and processed foods (e.g., sweets, cookies) are more rapidly absorbed and may have a greater detrimental effect than sugars from fruit or other natural sources [13], [45].

Our analysis in patients treated within a U.S. National Cancer Institute–sponsored clinical trial provided several advantages. All patients had lymph node–positive, non-metastatic cancer at study entry, reducing the impact of heterogeneity by disease stage. Treatment and patient follow-up were carefully prescribed in the trial, and the date and nature of cancer recurrence were prospectively recorded through regular detailed medical examinations. Detailed information on other potentially confounding prognostic variables was also prospectively collected at study entry. Finally, we updated dietary data to reflect changes in diet that may have occurred after patients had completed adjuvant therapy and recovered from treatment effects. Repeated dietary intake data served to reduce random measurement error, and any residual random error would likely lead to an underestimate of the true effect of exposures with outcome.

Nonetheless, we do recognize limitations within our analysis. Patients who enroll in randomized trials may differ from the population at large. However, the distribution of dietary intake and lifestyle factors in our cohort was consistent with other U.S. cohort studies. [46] Moreover, since the study included patients from community and academic centers throughout North America, our findings should reflect the general population.

We cannot completely exclude the possibility that increased sugar-sweetened beverage consumption may simply reflect other predictors of poor cancer prognosis. However, the effect of sugar-sweetened beverage was independent of other known or suspected prognostic factors, and our results remained largely unchanged when we further adjusted for other energy balance factors, including BMI, physical activity, Western and prudent pattern diets, and dietary glycemic load. Further, the detrimental effect of sugar-sweetened beverage intake persisted across strata of prognostic variables and dietary/lifestyle factors. Nevertheless, because of the observational nature of this study, we cannot exclude the possibility of residual and unmeasured confounding variable. Finally, while the original companion study for CALGB 89803 did pre-specify analyses of associations of dietary factors associated with energy balance and outcomes, caution should also be exercised when interpreting studies with multiple comparisons and P values>0.01. However, the point estimates are consistent across strata, and results are consistent with other analyses of energy balance in this cohort. [10], [22], [47], [48] Nonetheless, these results require confirmation in other cohorts.

We considered the possibility that patients with either occult cancer recurrences or other statistically significant poor prognostic characteristics may have increased their sugar-sweetened beverage intake as an alternative source of needed calories. To minimize this bias, we excluded recurrences or deaths within 90 days of FFQ completion. When we extended this restriction to 6 months, we continued to observe a deleterious association of sugar-sweetened beverage consumption on patient outcomes. Finally, because patients on this clinical trial underwent comprehensive staging at study entry and were followed with prescribed follow-up visits and testing, we would expect few patients to have undetected recurrences over extended periods.

Given that patients who consume sugar-sweetened beverages after cancer diagnosis may have consumed a similar diet before diagnosis, we cannot exclude the possibility that consumers of sugar-sweetened beverages develop biologically more aggressive colon cancers. Nonetheless, we did not observe a meaningful association between sugar-sweetened beverage intake and tumor-related characteristics associated with cancer recurrence.

In summary, this prospective analysis, imbedded in a clinical trial, suggests that increased sugar-sweetened beverage intake is associated with a significantly worse disease-free and recurrence-free survival for stage III colon cancer patients. Although our observational study does not provide conclusive evidence for causality, these findings support the potential role of sugar-sweetened beverage intake in colon cancer progression and potentially offer meaningful recommendations for clinical care. Further analyses in other cohorts of colon cancer survivors are needed to confirm these findings.

## References

[1] Giovannucci E (2003) Diet, body weight, and colorectal cancer: a summary of the epidemiologic evidence. J Womens Health (Larchmt) 12: 173–182. 12737716 10.1089/154099903321576574

[2] Moghaddam AA, Woodward M, Huxley R (2007) Obesity and risk of colorectal cancer: a meta-analysis of 31 studies with 70,000 events. Cancer Epidemiol Biomarkers Prev 16: 2533–2547. 18086756 10.1158/1055-9965.EPI-07-0708

[3] Samad AK, Taylor RS, Marshall T, Chapman MA (2005) A meta-analysis of the association of physical activity with reduced risk of colorectal cancer. Colorectal Dis 7: 204–213. 15859955 10.1111/j.1463-1318.2005.00747.x

[4] Chan AT, Giovannucci EL (2010) Primary prevention of colorectal cancer. Gastroenterology 138: 2029–2043 e2010. 10.1053/j.gastro.2010.01.057 PMC2947820 20420944

[5] Giovannucci E (2001) Insulin, insulin-like growth factors and colon cancer: a review of the evidence. J Nutr 131: 3109S–3120S. 11694656 10.1093/jn/131.11.3109S

[6] Kaaks R, Toniolo P, Akhmedkhanov A, Lukanova A, Biessy C, et al (2000) Serum C-peptide, insulin-like growth factor (IGF)-I, IGF-binding proteins, and colorectal cancer risk in women. J Natl Cancer Inst 92: 1592–1600. 11018095 10.1093/jnci/92.19.1592

[7] Schoen RE, Tangen CM, Kuller LH, Burke GL, Cushman M, et al (1999) Increased blood glucose and insulin, body size, and incident colorectal cancer. J Natl Cancer Inst 91: 1147–1154. 10393723 10.1093/jnci/91.13.1147

[8] Wei EK, Ma J, Pollak MN, Rifai N, Fuchs CS, et al (2005) A prospective study of C-peptide, insulin-like growth factor-I, insulin-like growth factor binding protein-1, and the risk of colorectal cancer in women. Cancer Epidemiol Biomarkers Prev 14: 850–855. 15824155 10.1158/1055-9965.EPI-04-0661

[9] Demark-Wahnefried W, Platz EA, Ligibel JA, Blair CK, Courneya KS, et al (2012) The role of obesity in cancer survival and recurrence. Cancer Epidemiol Biomarkers Prev 21: 1244–1259. 22695735 10.1158/1055-9965.EPI-12-0485 PMC3415558

[10] Meyerhardt JA, Niedzwiecki D, Hollis D, Saltz LB, Hu FB, et al (2007) Association of dietary patterns with cancer recurrence and survival in patients with stage III colon cancer. Jama 298: 754–764. 17699009 10.1001/jama.298.7.754

[11] Wolpin BM, Meyerhardt JA, Chan AT, Ng K, Chan JA, et al (2009) Insulin, the insulin-like growth factor axis, and mortality in patients with nonmetastatic colorectal cancer. J Clin Oncol 27: 176–185. 19064975 10.1200/JCO.2008.17.9945 PMC2645084

[12] Hu FB, Malik VS (2010) Sugar-sweetened beverages and risk of obesity and type 2 diabetes: epidemiologic evidence. Physiol Behav 100: 47–54. 20138901 10.1016/j.physbeh.2010.01.036 PMC2862460

[13] Schulze MB, Manson JE, Ludwig DS, Colditz GA, Stampfer MJ, et al (2004) Sugar-sweetened beverages, weight gain, and incidence of type 2 diabetes in young and middle-aged women. Jama 292: 927–934. 15328324 10.1001/jama.292.8.927

[14] Malik VS, Schulze MB, Hu FB (2006) Intake of sugar-sweetened beverages and weight gain: a systematic review. Am J Clin Nutr 84: 274–288. 16895873 10.1093/ajcn/84.1.274 PMC3210834

[15] Mozaffarian D, Hao T, Rimm EB, Willett WC, Hu FB (2011) Changes in diet and lifestyle and long-term weight gain in women and men. N Engl J Med 364: 2392–2404. 21696306 10.1056/NEJMoa1014296 PMC3151731

[16] Vartanian LR, Schwartz MB, Brownell KD (2007) Effects of soft drink consumption on nutrition and health: a systematic review and meta-analysis. Am J Public Health 97: 667–675. 17329656 10.2105/AJPH.2005.083782 PMC1829363

[17] Malik VS, Hu FB (2011) Sugar-sweetened beverages and health: where does the evidence stand? Am J Clin Nutr 94: 1161–1162. 21993436 10.3945/ajcn.111.025676 PMC3192470

[18] Andreyeva T, Chaloupka FJ, Brownell KD (2011) Estimating the potential of taxes on sugar-sweetened beverages to reduce consumption and generate revenue. Prev Med 52: 413–416. 21443899 10.1016/j.ypmed.2011.03.013

[19] Singh GM, Micha R, Katibzadeh S, Lim S, Ezzati M, et al.. (2013) 180,000 deaths worldwide may be associated with sugary soft drinks. Epidemiology and Prevention/Nutrition, Physical Activity and Metabolism 2013 New Orleans. pp. MP22.

[20] Saltz LB, Niedzwiecki D, Hollis D, Goldberg RM, Hantel A, et al (2007) Irinotecan fluorouracil plus leucovorin is not superior to fluorouracil plus leucovorin alone as adjuvant treatment for stage III colon cancer: results of CALGB 89803. J Clin Oncol 25: 3456–3461. 17687149 10.1200/JCO.2007.11.2144

[21] Zubrod C, Scheiderman M, Frei E (1960) Appraisal of methods for the study of chemotherapy in man: comparative therapeutic trial of nitrogen mustard and triethylene thiophosphoramide. J Chron Dis 11: 7–33.

[22] Meyerhardt JA, Sato K, Niedzwiecki D, Ye C, Saltz LB, et al (2012) Dietary glycemic load and cancer recurrence and survival in patients with stage III colon cancer: findings from CALGB 89803. J Natl Cancer Inst 104: 1702–1711. 23136358 10.1093/jnci/djs399 PMC3502194

[23] Willett WC, Reynolds RD, Cottrell-Hoehner S, Sampson L, Browne ML (1987) Validation of a semi-quantitative food frequency questionnaire: comparison with a 1-year diet record. J Am Diet Assoc 87: 43–47. 3794132

[24] Willett WC, Sampson L, Stampfer MJ, Rosner B, Bain C, et al (1985) Reproducibility and validity of a semiquantitative food frequency questionnaire. Am J Epidemiol 122: 51–65. 4014201 10.1093/oxfordjournals.aje.a114086

[25] Agriculture UDo (1989) Composition of foods - raw, processed, and prepared, 1963–1988. Agriculture handbook no. 8 series. Washington, D.C. Department of Agriculture, US Government Printing Office.

[26] Willett W, Stampfer MJ (1986) Total energy intake: implications for epidemiologic analyses. Am J Epidemiol 124: 17–27. 3521261 10.1093/oxfordjournals.aje.a114366

[27] Salvini S, Hunter DJ, Sampson L, Stampfer MJ, Colditz GA, et al (1989) Food-based validation of a dietary questionnaire: the effects of week- to-week variation in food consumption. Int J Epidemiol 18: 858–867. 2621022 10.1093/ije/18.4.858

[28] Meyerhardt JA, Heseltine D, Campos H, Holmes MD, Willett WC, et al (2005) Assessment of a dietary questionnaire in cancer patients receiving cytotoxic chemotherapy. J Clin Oncol 23: 8453–8460. 16293876 10.1200/JCO.2005.02.5460

[29] Meyerhardt JA, Fuchs C (2007) Cancer Recurrence and Survival Associated With Dietary Patterns in Stage III Colon Cancer-Reply Jama. 298: 2263. 10.1001/jama.298.19.2263-a 18029829

[30] Cox D (1972) Regression models and life tables. J R Stat Soc B 34: 187–220.

[31] Ainsworth BE, Haskell WL, Leon AS, Jacobs DR Jr, Montoye HJ, et al (1993) Compendium of physical activities: classification of energy costs of human physical activities. Med Sci Sports Exerc 25: 71–80. 8292105 10.1249/00005768-199301000-00011

[32] Theodoratou E, Farrington SM, Tenesa A, McNeill G, Cetnarskyj R, et al.. (2013) Associations between dietary and lifestyle risk factors and colorectal cancer in the Scottish population. Eur J Cancer Prev. 10.1097/CEJ.0b013e3283639fb8 23820601

[33] Zhang X, Albanes D, Beeson WL, van den Brandt PA, Buring JE, et al (2010) Risk of colon cancer and coffee, tea, and sugar-sweetened soft drink intake: pooled analysis of prospective cohort studies. J Natl Cancer Inst 102: 771–783. 20453203 10.1093/jnci/djq107 PMC2879415

[34] Malik VS, Popkin BM, Bray GA, Despres JP, Willett WC, et al (2010) Sugar-sweetened beverages and risk of metabolic syndrome and type 2 diabetes: a meta-analysis. Diabetes Care 33: 2477–2483. 20693348 10.2337/dc10-1079 PMC2963518

[35] Choi HK, Willett W, Curhan G (2010) Fructose-rich beverages and risk of gout in women. Jama 304: 2270–2278. 21068145 10.1001/jama.2010.1638 PMC3058904

[36] de Ruyter JC, Olthof MR, Kuijper LD, Katan MB (2012) Effect of sugar-sweetened beverages on body weight in children: design and baseline characteristics of the Double-blind, Randomized INtervention study in Kids. Contemp Clin Trials 33: 247–257. 22056980 10.1016/j.cct.2011.10.007

[37] Ebbeling CB, Feldman HA, Chomitz VR, Antonelli TA, Gortmaker SL, et al (2012) A randomized trial of sugar-sweetened beverages and adolescent body weight. N Engl J Med 367: 1407–1416. 22998339 10.1056/NEJMoa1203388 PMC3494993

[38] Qi Q, Chu AY, Kang JH, Jensen MK, Curhan GC, et al (2012) Sugar-sweetened beverages and genetic risk of obesity. N Engl J Med 367: 1387–1396. 22998338 10.1056/NEJMoa1203039 PMC3518794

[39] Pollak MN, Perdue JF, Margolese RG, Baer K, Richard M (1987) Presence of somatomedin receptors on primary human breast and colon carcinomas. Cancer Lett 38: 223–230. 2961436 10.1016/0304-3835(87)90218-7

[40] Watkins L, Lewis L, Levine A (1990) Characterization of the synergistic effect of insulin and transferrin and the regulation of their receptors on a human colon carcinoma cell line. Int J Cancer 45: 372–375. 2406206 10.1002/ijc.2910450227

[41] Koenuma M, Yamori T, Tsuruo T (1989) Insulin and insulin-like growth factor 1 stimulate proliferation of metastatic variants of colon carcinoma 26. Jpn J Cancer Res 80: 51–58. 2540132 10.1111/j.1349-7006.1989.tb02244.x PMC5917674

[42] Bjork J, Nilsson J, Hultcrantz R, Johansson C (1993) Growth-regulatory effects of sensory neuropeptides, epidermal growth factor, insulin, and somatostatin on the non-transformed intestinal epithelial cell line IEC-6 and the colon cancer cell line HT 29. Scand J Gastroenterol 28: 879–884. 7505479 10.3109/00365529309103129

[43] Mattes RD (1996) Dietary compensation by humans for supplemental energy provided as ethanol or carbohydrate in fluids. Physiol Behav 59: 179–187. 8848479 10.1016/0031-9384(95)02007-1

[44] DiMeglio DP, Mattes RD (2000) Liquid versus solid carbohydrate: effects on food intake and body weight. Int J Obes Relat Metab Disord 24: 794–800. 10878689 10.1038/sj.ijo.0801229

[45] Stanhope KL, Schwarz JM, Keim NL, Griffen SC, Bremer AA, et al (2009) Consuming fructose-sweetened, not glucose-sweetened, beverages increases visceral adiposity and lipids and decreases insulin sensitivity in overweight/obese humans. J Clin Invest 119: 1322–1334. 19381015 10.1172/JCI37385 PMC2673878

[46] Michaud DS, Fuchs CS, Liu S, Willett WC, Colditz GA, et al (2005) Dietary glycemic load, carbohydrate, sugar, and colorectal cancer risk in men and women. Cancer Epidemiol Biomarkers Prev 14: 138–147. 15668487

[47] Meyerhardt JA, Heseltine D, Niedzwiecki D, Hollis D, Saltz LB, et al (2006) Impact of physical activity on cancer recurrence and survival in patients with stage III colon cancer: findings from CALGB 89803. J Clin Oncol 24: 3535–3541. 16822843 10.1200/JCO.2006.06.0863

[48] Meyerhardt JA, Niedzwiecki D, Hollis D, Saltz LB, Mayer RJ, et al (2008) Impact of body mass index and weight change after treatment on cancer recurrence and survival in patients with stage III colon cancer: findings from Cancer and Leukemia Group B 89803. J Clin Oncol 26: 4109–4115. 18757324 10.1200/JCO.2007.15.6687 PMC2654367

